# Prospective Associations between Popularity, Victimization, and Aggression in Early Adolescence

**DOI:** 10.1007/s10964-020-01248-4

**Published:** 2020-05-12

**Authors:** Sarah T. Malamut, Tana Luo, David Schwartz

**Affiliations:** 1 INVEST Research Flagship, Department of Psychology, University of Turku, Turku, Finland; 2 Eating Disorders Center for Treatment and Research, University of California, San Diego, CA USA; 3 Department of Psychology, University of Southern California, Los Angeles, CA USA

## Abstract

Recent research has highlighted an understudied phenomenon in the peer victimization literature thus far: the overlap between high status (i.e., popularity) and victimization. However, the research on this phenomenon has primarily been cross-sectional. The current investigation uses a longitudinal design to address two questions related to high-status victims. First, the present study examined prospective associations between popularity and two forms of indirect victimization (reputational victimization and exclusion). Second, this study examined elevated aggression as a consequence of high-status youth’s victimization (using self- and peer- reports of victimization). Participants were 370 adolescents (*M*_age_ = 14.44, range = 14.00–16.00; 56.5% girls) who were followed for 1 year. Both high and low levels of popularity were prospectively associated with reputational victimization. Moreover, popularity moderated the association between self-reported indirect victimization (but not peer-reported indirect victimization) and aggression. The results help build toward a more comprehensive understanding of both victimization and aggression in adolescence. Findings are discussed in terms of implications for a cycle of aggression in youth and the lowered effectiveness of bullying interventions in adolescence.

## Introduction

In adolescence, there is a consistent and robust association between popularity and aggression (Cillessen and Mayeux 2004). Paradoxically, there is also emerging evidence that popular youth are not just perpetrators but are also victims of aggression (e.g., Dawes and Malamut 2018). However, due to a dearth of longitudinal studies, many questions still remain regarding the experiences of high-status victims. The purpose of the current study was to address two distinct but related questions regarding this phenomenon. The first goal was to understand whether popular youth are more likely to experience specific subtypes of indirect victimization (i.e., reputational victimization, exclusion) over time. The second goal was to examine how popular youth’s own perceptions of being victimized are related to subsequent indirect aggression.

### Popularity and Victimization

High levels of popularity are typically thought of as protective against victimization, as popularity is an indicator of social success. Nevertheless, there are several explanations for why popular youth may be targeted by peers (see Dawes and Malamut 2018 for a review). Through the lens of evolutionary psychology and social dominance perspectives, the function of aggression is to gain power and access to valued resources, and to improve one’s position in the social hierarchy (e.g., Volk et al. 2012). Importantly, resources that are valued in adolescence (e.g., social centrality; Dawes and Malamut 2018) are also finite, and not everyone in the peer group can reach the top of the social hierarchy. Insofar as youth use aggression to gain social rewards and/or to climb the social ladder, perpetrators may choose to target popular peers who currently have access to the desired resources and social position (i.e., instrumental targeting: Faris and Felmlee 2014). Moreover, popular youth may be targeted by other popular peers who see the target as potential social competition (e.g., Andrews et al. 2017).

### Subtypes of Victimization

Successfully identifying popular victims is, in part, dependent on the type of victimization being measured. For example, it may be difficult to identify popular victims with peer-reports of who is bullied or picked on because popular youth, due to their social success, likely do not have a reputation as youth who are often bullied. Furthermore, given that popular adolescents have an assortment of social resources and are dominant in the peer group, youth may be more likely to use some forms of aggression (e.g., covert or “behind-the-back” aggression) against popular peers than others to avoid the risk of a direct confrontation (Dawes and Malamut 2018). Indeed, in a review of the extant literature examining high-status victims, Dawes and Malamut (2018) found less support that popularity was linked to victimization via overt or direct forms of aggression. Instead, popular youth were more likely to be targeted with indirect or relational aggression. As such, the present study will focus on indirect forms of aggression and victimization.

It is important to note that there are ongoing debates over how to refer to nonphysical aggression that may involve covert behaviors or manipulation of peer relationships to hurt the victim (Voulgaridou and Kokkinos 2015). This type of aggression has been referred to as indirect aggression (Björkqvist et al. 1992), relational aggression (Crick and Grotpeter 1995), and social aggression (Underwood et al. 2001). However, in a comprehensive review, Archer and Coyne (2005) found relatively few conceptual or empirical differences between indirect aggression, relational aggression, and social aggression. Consistent with their recommendation, the current study refers to these behaviors as indirect aggression/victimization.

Indirect victimization is often treated as a homogenous entity in the extant literature, despite support that subtypes of indirect victimization occur at different rates and are differentially related to other characteristics (e.g., Closson et al. 2017; Prinstein and Cillessen 2003). For example, a typical assumption is that the same youth who are the victims of rumors or disparaging gossip (i.e., reputational victimization) are also likely to experience exclusion. However, there are several reasons why high levels of popularity may be associated with reputational victimization, but not exclusion. A key feature of gossip or rumor spreading is that the perpetrator is able to easily conceal his/her identity (Xie et al. 2005). Whereas some peers may hesitate to aggress against a popular peer out of fear of retaliation, reputational aggression can be a low-risk way of damaging a social competitor’s social standing (Prinstein and Cillessen 2003). On the other hand, it is likely harder to successfully exclude popular peers from activities, given their social resources and centrality in the peer group. It is important to note that popular youth may still be excluded from activities (e.g., within their friendships: Closson and Watanabe 2018); however, it may be unbeknownst to the broader peer group. Due to popular youth’s social success and centrality in the peer group, their classmates may be unlikely to view them as excluded or neglected.

Indeed, there is some evidence of positive, concurrent associations between popularity and reputational victimization, but less support for positive links between popularity and experiences of exclusion (Closson et al. 2017). Yet, measures of reputational victimization are often combined with measures of exclusion, despite that these forms of aggression serve different functions and have unique associations with popularity (Prinstein and Cillessen 2003). Thus, combining measures of reputational victimization and exclusion may make it difficult to identify victims with high levels of popularity, as any positive association between popularity and reputational victimization may be suppressed by including exclusion. Moreover, despite support of concurrent associations between popularity and reputational victimization, prospective relations have not been studied. Therefore, it is still unclear whether popularity may actually be a risk factor for certain types of victimization.

Both high and low popularity may be risk factors for reputational victimization. Reputational aggression can be used to target a high-status peer (e.g., social competition) or a low-status peer (e.g., choosing an easy target; Malamut et al. 2018). Consistent with past research, a curvilinear association was expected between popularity and reputational victimization (Prinstein and Cillessen 2003). High (and low) levels of popularity were expected to be associated with high levels of reputational victimization over time, as youth may use this form of aggression in attempts to damage popular youth’s social standing or reputation, or against low-status youth to establish social norms (e.g., (non)acceptable behaviors; Prinstein and Cillessen 2003). On the other hand, there are aspects of popularity (e.g., social resources, centrality) that should generally be protective against other types of aggression, such as being excluded or neglected. Therefore, high popularity was expected to be negatively associated with being excluded over time.

### Victimization, Popularity, and Aggression

Not only may popular youth be at elevated risk for certain types of victimization, but their experiences being victimized likely also contributes to a cycle of aggression in the peer group. Victimization by peers is a risk factor for future aggression (e.g., Cooley et al. 2017). Youth who have experienced victimization may be at elevated risk for aggression, either in retaliation or to defend themselves against more victimization (e.g., Yeung and Leadbeater 2007). The understudied association between victimization and popularity could be related to this effect, such that popular youth react to their (perceived) mistreatment by peers. That is, popular adolescents, who already enjoy the benefits of social status (e.g., social resources and visibility), may be particularly sensitive to challenges to their social standing (i.e., victimization), and subsequently engage in behaviors intended to maintain status (e.g., aggression).

Indeed, Faris and Felmlee (2014) found that the association between victimization and adverse outcomes (e.g., anxiety, depression, anger) was magnified for socially central adolescents, and speculated that this was because these adolescents had “more to lose” (e.g., prominence and social resources). Moreover, Ferguson et al. (2016) found some support that the bidirectional association between victimization and aggression was moderated by social status. They found that popular girls who were victimized became more aggressive 7 months later. Taken together, these findings support that the association between victimization and aggression may be stronger for popular youth than their less popular peers, as popular youth may become more aggressive in an attempt to protect their status.

Therefore, in addition to examining main effect relations between victimization and popularity, the current study also considered the potential for the overlap of victimization and popularity to have a role in risk for future perpetration of aggression. Whereas Ferguson et al. 2016 used peer nominations to assess victimization, self-perceived victimization is likely particularly pertinent for subsequent aggression, especially when an adolescent is popular. There are several reasons to expect different associations for self- and peer-reported victimization. If elevated aggression is a potential consequence of victimization due to retaliation or a desire to protect oneself from future victimization (e.g., Yeung and Leadbeater 2007), then this is likely driven by youth perceiving themselves as victimized or threatened. That is, if they do not see themselves as victimized, then there is no reason to retaliate or defend themselves. Therefore, youth’s feelings of having their status threatened or being victimized (i.e., self-reported victimization) was expected to impact future aggression, especially for popular youth.

Specifically, self-reported victimization was expected to predict increases in aggression at high levels of popularity. However, given previous findings (e.g., Ferguson et al. 2016), peer-reports of victimization were also included as a comparison. Furthermore, it is important to consider both self- and peer-reports of victimization given past research demonstrating that the associations of victimization differ between informants (e.g., Scholte et al. 2013).

Aggression can be used to target a potential social competitor or, conversely, to demonstrate one’s dominance by picking on a weaker peer (e.g., Volk et al. 2014). As such, adolescents who are high in popularity but feel threatened (via victimization) could try to get revenge on their aggressor or could re-establish their dominance by targeting a weak classmate. Whereas reputational victimization and exclusion were expected to be differently associated with popularity, there were no expected differences regarding popularity and the use of reputational aggression versus exclusion toward others. In fact, youth who are popular often use both forms of aggression against their peers (Prinstein and Cillessen 2003). Closson and Hymel (2016) found popular adolescents, as compared to unpopular youth, used higher levels of indirect aggression and direct aggression against their peers. Unlike their peers with less power, youth with elevated popularity have the social resources and support to enact any form of aggression towards their classmates. As there were not any a priori hypotheses to expect self-perceived victimization to be differentially related to different forms of indirect aggression, the current study examined overall levels of indirect aggression.

## The Current Investigation

Despite growing evidence that youth with high status are also targets of aggression (e.g., Dawes and Malamut 2018), previous research often does not identify these types of victims, perhaps due to the form of victimization assessed and/or because their peers do not view them as victims. High-status victims are an understudied group of victims whose experiences may be associated with subsequent aggression (Dawes and Malamut 2018). First, the present study examined if popularity was differentially related to changes in reputational victimization and exclusion over time. High popularity was expected to predict increases in reputational victimization over time, but decreases in being excluded over time. Second, the current study examined whether popularity moderated the association between (self-reported) indirect victimization and subsequent aggression. These questions are particularly pertinent in early adolescence, as youth increasingly prioritize popularity (LaFontana and Cillessen 2010) and therefore may use aggression more strategically in an attempt to gain status (e.g., by targeting popular peers). Whereas the subtype of victimization was expected to be essential when using peer reports to identify victims with high status, there was not a similar hypothesis for the association between self-reported indirect victimization and aggression. In other words, there was no a priori reason to expect that self-reports of being a rumor victim would be differentially related to aggression than self-reports of being excluded, as any form of victimization would result in feeling threatened or self-perceptions as victimized. Therefore, self-reported indirect victimization (regardless of subtype) was hypothesized to predict increases in indirect aggression, particularly for youth with high levels of popularity.

## Methods

### Participants and Procedure

This study was completed in collaboration with a high school in the greater Los Angeles area. In spring 2016 (T1), 659 9th graders were invited to participate and 413 received positive parental consent. Participants at T1were 379 adolescents (*M*_*age*_ = 14.4; 56.2% girls) in the 9th grade who assented to participate and were not absent during data collection. The ethnic/racial composition of the sample was 29.3% Latino/Hispanic, 26.9% White, 10.0% Asian/Pacific Islander, 2.1% African American, 0.3% American Indian, 27.7% mixed, and 3.7% not classified. In spring 2017 (T2), a follow-up data collection was completed when participants were in 10th grade. Of the participants at T1, we retained almost the full sample (*n* = 374). Of these, 4 students were excluded from analyses for missing data. Little (1988) MCAR test indicated that the missing values were missing completely at random, *χ*^2^ = 6.77, df = 12, *p* = 0.87. The remaining 370 participants (*M*_*age*_ = 14.4; 56.5% girls) had full data for the items relevant to the current study.

At each wave, trained graduate and undergraduate research assistants administered the measures to participants. The research assistants read out loud the standardized instructions to the participants and reiterated the confidentiality of their responses. Research assistants informed participants that they could stop participating at any time, and were also available to answer any questions. This project was approved by the university’s Internal Review Board (IRB # UP-15-00579-CR002 “School Adjustment”).

### Measures

#### Victimization (self-report)

Participants completed a 10-item self-report questionnaire that assesses youth’s experiences of indirect and direct victimization. For the current investigation, we focused on five items pertaining to indirect victimization (e.g., “try to keep others from liking you”). As noted earlier, our expectation was that any perception of victimization (regardless of subtype) would be associated with elevated aggression for popular youth. Accordingly, analyses were conducted using the averaged participants’ scores on the five items. In addition to the theoretical basis for averaging the five items, there was also empirical support as the items had high reliability (Cronbach’s α = 0.85) and an exploratory factor analysis indicated a one factor solution (62.9% of the variance explained with factor loadings from 0.67 to 0.84).

#### Victimization (peer nomination)

Peer nominations were also used to assess youth’s experiences with victimization. Participants were given a random list of approximately 50 participating grademates (e.g., Bellmore et al. 2010). Participants then indicated which peers fit a series of descriptors, with unlimited nominations. Nominations were summed and then standardized within list. Given the goal to differentiate between victimization experiences (e.g., Closson et al. 2017; Prinstein and Cillessen 2003), separate indices were included for reputational victimization (“students who get mean things said about them”) and exclusion (“students that get left out of activities, excluded, or ignored when other students are trying to hurt their feelings”).

#### Aggression (peer nominations)

Youth’s indirect aggression was measured using peer nominations, using the same procedure described above. Participants were asked to nominate “students that gossip about other students” and “students that try to be mean to other students by ignoring them or excluding them” (*r* = 0.75, *p* < 0.0001 at T1; *r* = 0.70, *p* < 0.0001 at T2). The nominations for each item was summed and the average of the two items was standardized within list.

#### Popularity (peer nominations)

Peer nominations were also used to assess participants’ popularity. Youth nominated their peers who were “most popular” and “least popular”. Standardized nominations were calculated for each item using the same method described above, and a difference score was computed (most popular − least popular) and restandardized (Cillessen and Marks 2011).

## Results

### Overview

The present study sought to examine the longitudinal associations of popularity, indirect victimization (reputational victimization, exclusion), and indirect aggression. Analyses were conducted to examine how the predictors at T1 were associated with the outcome at T2, with the outcome variable at T1 controlled for in each set of analyses. Before the primary analyses, bivariate correlations were examined. The primary research aims were investigated by conducting linear regressions in R. In each set of analyses, gender was explored as a possible moderator. Although potential gender differences were not a primary focus of the current study, the exploratory analysis was conducted given mixed findings that girls may be more likely to use indirect aggression than boys (Archer 2004).

### Descriptive Statistics

Means, standard deviations, and correlations among study variables are shown in Table 1. All continuous variables were mean centered. Girls reported higher levels of self-reported victimization at T1 and indirect aggression at both time points than boys. There were no significant gender differences in peer-nominated victimization (i.e., exclusion, reputational victimization) or popularity.

**Table 1:** Correlations, means, and independent sample t-tests

	1	2	3	4	5	6	7	8	*M* (SD)	*M* (SD)_boys_ (*n* = 161)	*M* (SD)_girls_ (*n* = 209)	*t*
1. Self-reported victimization (T1)	–								1.62 (0.72)	1.46 (0.56)	1.73 (0.81)	−3.78***
2. Exclusion (T1)	0.07	–							0.01 (1.01)	0.11 (1.07)	−0.07 (0.95)	1.67
3. Reputational victimization (T1)	0.27***	0.51***	–						−0.01 (0.98)	−0.01 (0.92)	−0.01 (1.04)	−0.02
4. Indirect aggression (T1)	0.27***	−0.03	0.50***	–					−0.03 (0.93)	−0.23 (0.71)	0.12 (1.04)	−3.79***
5. Popularity (T1)	0.17**	−0.34***	0.10*	0.62***	–				−0.03 (0.97)	−0.13 (0.95)	0.05 (0.98)	−1.81
6. Exclusion (T2)	0.04	0.53***	0.33***	−0.07	−0.28***	–			−0.01 (0.95)	0.11 (1.04)	−0.08 (0.86)	1.92
7. Reputational victimization (T2)	0.19***	0.38***	0.64***	0.41***	0.12*	0.47***	–		0.01 (0.99)	0.03 (1.01)	−0.02 (0.98)	0.46
8. Indirect aggression (T2)	0.30***	−0.04	0.40***	0.74***	0.58***	−0.01	0.51***	–	−0.02 (0.96)	−0.29 (64)	0.19 (1.10)	−5.30***

*p < 0.05, **p < 0.01, ***p < 0.001

Self-reported victimization (T1) was positively associated with reputational victimization (T1 and T2), indirect aggression (T1 and T2), and popularity (T1). Moreover, popularity (T1) was positively related to indirect aggression (T1 and T2) and reputational victimization (T1 and T2), and negatively related to exclusion (T1 and T2). Indirect aggression and reputational victimization, but not exclusion, were positively correlated at both time points. Indirect aggression and both forms of peer nominated victimization were stable from T1 to T2.

### Prospective Associations between Popularity and Peer-Nominated Victimization

The first goal was to test the hypothesis that high levels of popularity would be associated with high levels of reputational victimization, but low levels of exclusion, over time. Separate linear regressions were conducted for reputational victimization and exclusion. In each model, the form of victimization at T2 was predicted by popularity and T1 victimization, while controlling for gender (Table 2).

**Table 2:** Predicting T2 peer-nominated victimization from T1 popularity

	Reputational victimization T2		Exclusion T2	
	*b*	SE	*b*	SE
Gender	−0.07	0.08	−0.09	0.08
Reputational victimization T1	0.56***	0.04	–	–
Exclusion T1	–	–	0.46***	0.04
Popularity (linear term) T1	0.04	0.04	−0.10*	0.05
Popularity (quadratic term) T1	0.13***	0.03	–	–

*p < 0.05, **p < 0.01, ***p < 0.001

For the model predicting T2 reputational victimization, a quadratic popularity term was included, given the hypothesis that there would be a curvilinear effect of popularity (i.e., an association at low and high levels of popularity). The overall model was significant, *F*(4, 365) = 71.46, *p* < 0.001, *R*^2^ = 0.43. The quadratic popularity term was significant (*β* = 0.19, *p* < 0.001), but the linear popularity term was not (*β* = 0.04, *p* = 0.272). As indicated by the positive coefficient term, both high and low levels of T1 popularity were associated with elevated T2 reputational victimization. Next, popularity as a predictor of exclusion was tested. The model predicting T2 exclusion was significant, *F*(3, 366) = 49.78, *p* < 0.001, *R*^2^ = 0.28. There was a negative association between T1 popularity and exclusion at T2 (*β* = −0.10, *p* = 0.028). There were no significant gender differences in either model.

### Self-Reported Victimization, Popularity, and Aggression

To examine whether self-reported indirect victimization predicts increases in indirect aggression, along with whether this association is moderated by popularity, we conducted linear regressions. As before, separate models were conducted for reputational victimization and exclusion. For each set of analyses, the main effects of self-reported victimization, popularity, and peer-nominated victimization at T1 on indirect aggression at T2 were first examined, while controlling for gender and indirect aggression at T1. The model including reputational victimization was significant, *F*(5, 364) = 109.51, *p* < 0.001, *R*^2^ = 0.60. Indirect aggression was stable from T1 to T2 (*β* = 0.53, *p* < 0.001). High levels of self-reported victimization (*β* = 0.07, *p* = 0.045), reputational victimization (*β* = 0.10, *p* = 0.017), and popularity (*β* = 0.22, *p* < 0.001) were all independently associated with T2 indirect aggression (Table 3a, Model 1). The model including exclusion revealed a similar pattern of findings, *F*(5, 364) = 107.63, *p* < 0.001, *R*^2^ = 0.59 (see Table 3b, Model 1), with one exception. Exclusion did not predict indirect aggression (*β* = 0.05, *p* = 0.168).

**Table 3:** Predicting T2 indirect aggression from T1 victimization and T1 popularity

Panel A				
	Model 1		Model 2	
	*b*	SE	*b*	SE
Gender	0.23***	0.07	0.23***	0.07
Indirect aggression T1	0.53***	0.05	0.53***	0.05
Popularity T1	0.22***	0.04	0.21***	0.04
Self-reported victimization T1	0.09*	0.05	0.09*	0.05
Reputational victimization T1	0.10*	0.04	0.08^†^	0.04
Popularity × Self-reported victimization			0.12**	0.05
Popularity × Reputational victimization			0.00	0.03
**Panel B**				
	**Model 1**		**Model 2**	
	***b***	**SE**	***b***	**SE**
Gender	0.22***	0.07	0.22***	0.07
Indirect aggression T1	0.59***	0.05	0.57***	0.05
Popularity T1	0.21***	0.05	0.22***	0.05
Self-reported victimization T1	0.11*	0.05	0.10*	0.05
Exclusion T1	0.05	0.04	0.03	0.04
Popularity × Self-reported victimization			0.13**	0.04
Popularity × Exclusion			−0.03	0.03

*n* = 370. In Panel A, the model included peer-nominated reputational victimization. In Panel B, the model included peer-nominated exclusion

*p < 0.05, **p < 0.01, ***p < 0.001, ^†^*p* < 0.10

### The Moderating Role of Popularity

**Fig. 1:**
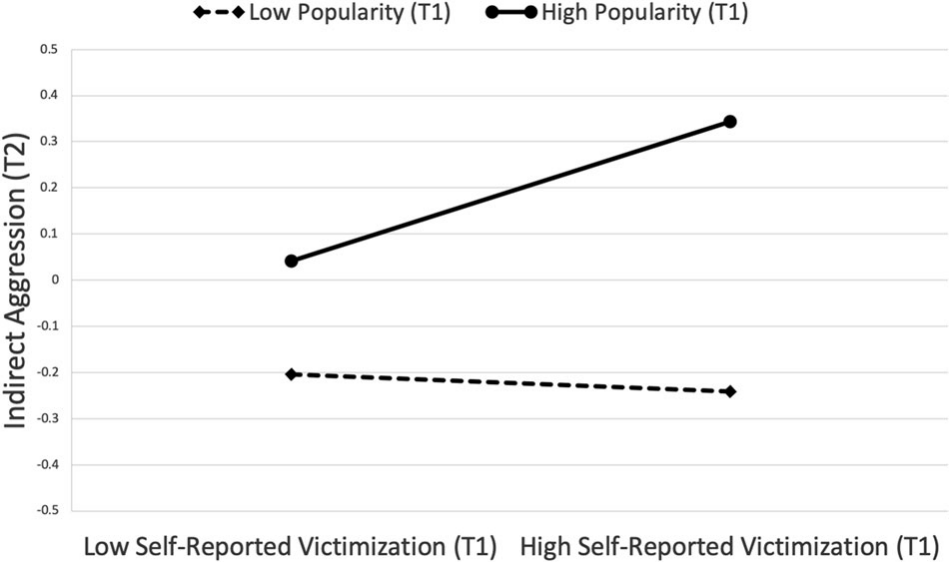
T1 popularity moderates the association between T1 self-reported victimization and T2 indirect aggression

Next, T1 popularity was examined as a moderator of the association between T1 self-reported victimization and T2 indirect aggression. In this model, two-way interactions were added between self-reported victimization and popularity, and reputational victimization and popularity, *F*(7, 362) = 80.46, *p* < 0.001, *R*^2^ = 0.60 (see Table 3a, Model 2). The effect of self-reported victimization on indirect aggression was qualified by popularity, (*β* = 0.09, *p* = 0.008). For participants with low popularity at T1, self-reported indirect victimization was not associated with indirect aggression at T2 (simple slopes test, *t* = −0.40, *p* = 0.69, Fig. 1). However, at high levels of T1 popularity, self-reported indirect victimization was associated with high T2 indirect aggression (simple slopes test, *t* = 3.29, *p* = 0.001). The two-way interaction between popularity and peer-reported victimization was not significant. There was also not a significant gender moderation. The same pattern of findings emerged when including exclusion in the model (see Table 3b, Model 2)^1^.

## Discussion

Recent research has emphasized the need to further examine the overlap between high popularity and victimization for a more comprehensive understanding of who is at risk for victimization and of the consequences of victimization (Dawes and Malamut 2018). Although there is growing evidence of a concurrent, positive association between popularity and victimization, it is not yet clear whether popularity predicts elevated victimization over time. It is essential to better understand popular youth’s experiences being victimized, as it may perpetuate a cycle of aggression in the peer group (Dawes and Malamut 2018). The findings shed light on how popularity and different forms of indirect victimization (i.e., reputational victimization and exclusion) are related over time. The findings also suggest that an overlap in self-perceived victimization and popularity play an important role in predicting future aggression.

### Popularity and Victimization

The first goal of the current investigation was to examine the prospective associations between popularity and two forms of indirect victimization. Consistent with past findings, high levels of popularity were associated with reputational victimization but not exclusion (e.g., Closson et al. 2017; Prinstein and Cillessen 2003). Interestingly, the association between indirect aggression and reputational victimization was about as strong at both time points (*rs* > 0.50) as the relation between reputational victimization and exclusion (*rs* > 0.47). Taken together, these findings support that indirect victimization should not be treated as a homogenous experience, and that there are differential associations across specific types of indirect victimization. As an extension beyond existing cross-sectional analyses, the results demonstrated that high popularity also predicted increases in reputational victimization over time.

This study supports the growing evidence that high popularity and victimization are not mutually exclusive, despite that victims of aggression have traditionally been thought to have low status (Dawes and Malamut 2018). Moreover, the results indicate that not only are popular youth victimized more than was previously thought, but that high popularity is actually a risk factor for certain types of victimization. This finding also suggests that one reason high-status victims may not be identified is because of lack of specificity in measures. Reputational victimization may be more often used against popular youth than other forms of aggression as it is easier for the perpetrator to hide his/her identity (e.g., Closson et al. 2017). On the other hand, popular adolescents have social resources and power, which makes it more difficult for their peers to exclude them from activities.

Along with lack of specificity in measures, the informant of victimization is another factor that is associated with the identification of high-status victims (Dawes and Malamut 2018). Past research suggests that the concordance between self- and peer- reports of victimization is typically low (Dawes et al. 2017; Scholte et al. 2013). However, studies examining the overlap between self- and peer- reports of victimization typically use peer nominations of general bullying (e.g., which classmates are bullied or picked on; Dawes et al. 2017; Scholte et al. 2013). High-status adolescents may not be nominated by classmates as a victim of general bullying due to their social success. As the results of the current study indicate, popularity was positively associated with self-reported victimization, but only positively associated with a specific form of peer nominated victimization (reputational victimization). Therefore, part of the discrepancy between self- and peer- reports may be because high-status victims perceive themselves to be victimized (self-reported victimization) but are not viewed by their classmates as a victim (peer-reported victimization). As self-reports and peer nominations are the two most common ways of identifying victims (Casper et al. 2015), it is crucial to understand factors that contribute to their low agreement. The current investigation suggests that high-status victims may be an understudied group related to the disagreement in informants. Indeed, past research using person-centered analyses have found that self-identified victims do not have the same social difficulties as other types of victims (Scholte et al. 2013). For example, a recent study by Dawes et al. (2019) found that teachers perceived self-identified victims to be more popular than other victims. Future research should further test the role of high-status victims in the discrepancy between self- and peer- reports of victimization.

### Self-Reported Victimization, Popularity, and Aggression

The second goal of the current study was to investigate whether the link between victimization and popularity was longitudinally related to elevated indirect aggression. As hypothesized, the association between self-reported victimization and subsequent aggression was moderated by popularity. At high levels of popularity, self-reported victimization predicted higher levels of indirect aggression over time. Notably, there was not a similar effect for peer nominated victimization. A potential limitation of past research utilizing both self- and peer-reports is shared method variance. In other words, these studies (e.g., Crick and Bigbee 1998; Graham and Juvonen 1998) found that self-reported victimization is more strongly associated with other self-reported difficulties (e.g., internalizing problems), whereas peer-reported victimization is more strongly associated to peer-reported difficulties, such as social problems (Scholte et al. 2013). In the current study, high levels of self-reported, but not peer-reported, victimization was associated with high levels of *peer*-reported aggression.

The current investigation suggests that popular youth’s victimization is a risk factor for subsequent indirect aggression. If youth report victimization and have social resources (i.e., popularity), then this contributes to increases in aggression. Given popular youth’s elevated influence (Dijkstra and Gest 2015), their subsequent increases in aggression may influence how aggression is viewed by the peer group. Dijkstra et al. (2008) found that bullying was viewed less negatively in classrooms with higher levels of bullying by popular youth. Moreover, behaviors that youth believe are associated with status are viewed positively, and youth may emulate these behaviors to gain status (e.g., Dijkstra et al. 2013). As such, popular youth’s increases in aggression may contribute to aggression being viewed as acceptable, and could even contribute to classmates becoming more aggressive. Therefore, popular youth’s victimization may also perpetuate a cycle of aggression in the broader peer group.

Furthermore, high-status youth’s experiences being victimized may help explain why current bullying interventions are less effective in adolescence and for popular bullies. Bullying interventions are typically less effective (or even ineffective) in adolescence, as compared to childhood (e.g., Yeager et al. 2018). Even at ages when bullying interventions have had more success (e.g., in childhood), they are less successful at reducing the bullying behaviors of popular youth than youth with average or low popularity (Garandeau et al. 2014). Bullying is a goal-directed behavior that is intended to provide the perpetrators with social benefits (e.g., Ellis et al. 2016). Aggression and social status are deeply intertwined, particularly in adolescence (e.g., Schwartz and Gorman 2011), which may contribute to the decreased effectiveness of bullying interventions with both older youth as well as younger, popular youth. In other words, popular youth may be particularly reluctant to decrease their aggression if they feel their status is being threatened via victimization.

### Strengths, Limitations, and Future Directions

The current study has many methodological and theoretical strengths. First, it underscores the benefit of highly specific peer nomination items for identifying subsets of victims. Second, it highlights an understudied subset of victims of aggression: victims with high status. Third, it built on past cross-sectional research to examine how popularity and victimization are associated over time. Lastly, it draws attention to a concerning pattern: self-perceived victimization predicts increased aggression over time, especially for popular adolescents.

Still, there were limitations that should be noted. There are other factors besides popularity that may be important in understanding the link between self-perceived victimization and aggression. For example, recent research has highlighted the role of popularity goal (i.e., how much one strives to be popular) in moderating the relation between popularity and aggression (Dawes and Xie 2014). It is possible that only popular self-identified victims who also value being popular will show increases in aggression. Future research should examine how social goals influence this association.

Although there were theoretical reasons to focus on the associations between popularity, indirect aggression, reputational victimization and exclusion, it is also important to consider other forms of victimization (e.g., Card and Hodges 2008). A sensitivity analysis, however, indicated that the findings replicated when using a combined measure of indirect and direct self-reported victimization (see Footnote 1). Moreover, while the current study focused on aggression, it is also important to understand how high-status youth’s victimization impacts their psychosocial adjustment (e.g., lowered self-esteem). Popularity is generally considered protective against internalizing problems (e.g., Litwack et al. 2012); however, this may not be true for popular youth who feel victimized.

The results of this study suggest that high-status victims may be an understudied group that is related to the low concordance between self- and peer- reports of victimization. Despite the strengths of the current investigation, the variable-centered analyses used do not allow a direct examination of whether a subset of self-identified victims are in fact youth with high status. Future research should use person-centered analyses to assess whether some self-identified victims have high popularity and aggression. This line of inquiry could inform whether the discordance between self-reports and peer nominations is partially driven by high-status victims.

Of note, the findings in this study were not moderated by gender, suggesting that the associations between victimization, popularity, and aggression may be largely the same for boys and girls. Nevertheless, it may be too early in this line of inquiry to dismiss potential gender differences. For example, the gender composition of perpetrators and victims in a bully-victim dyad appears to play an important role in the distribution of status within bully-victim dyads (e.g., Rodkin and Berger 2008; Sainio et al. 2012). Future research would benefit from examining the consequences of high-status youth’s victimization using a dyadic framework that accounts for perpetrators’ and victims’ individual characteristics (e.g., gender).

## Conclusion

The current investigation sheds light on some of the unanswered questions regarding high-status victims. The findings suggest not only that high popularity can be a risk factor for certain types of victimization, but that the overlap between high popularity and self-reported victimization is associated with elevated aggression. The present study supports using measures of victimization with high specificity, as reputational victimization, but not exclusion, was positively associated with popularity. Not using highly specific measures of victimization may preclude the identification of high-status victims, particularly in adolescence when youth may be more strategic with their aggression (e.g., targeting popular peers). Given that the positive association between self-reported victimization and aggression was magnified for youth with high levels of popularity, it is essential for future research to account for high-status victims. High-status victims’ experiences appear to be an integral piece to understanding cycles of aggression in the peer group; further research is needed to identify whether high-status victims contribute to the lack of success of bullying interventions in adolescence. The findings of the current study highlight the need to investigate popular youth’s experiences of victimization for a comprehensive understanding of both victimization and aggression in the peer group.

## Footnotes

1 We conducted sensitivity analyses, replicating our analyses with a combined measure of direct and indirect self-reported victimization, *F*(7, 362) = 78.83, *p* < 0.001, *R*^2^ = 0.60. Again, there was a significant interaction between self-reported victimization and popularity (*β* = 0.07, *p* = 0.047).
